# Syndrome de Sturge Weber associé au glaucome: à propos d'un cas

**DOI:** 10.11604/pamj.2014.18.338.4267

**Published:** 2014-08-27

**Authors:** Fatima Zohra El Meriague, Rajae Daoudi

**Affiliations:** 1 Université Mohamed V, Souissi, Service d'Ophtalmologie A de l'Hôpital des Spécialités, Centre Hospitalier Universitaire, Rabat, Maroc

**Keywords:** Syndrome de Sturge Weber, glaucome, exophtalmie, Sturge Weber syndrome, glaucoma, exophthalmos

## Image en medicine

Nous rapportons le cas d'un patient de 20 ans, sans antécédent pathologique particulier, qui présente depuis la naissance un angiome cutané facial, une baisse de l'acuité visuelle depuis 3 ans ainsi qu'une exophtalmie d'installation progressive de l'œil droit. L'examen ophtalmologique a montré une AV à 2/10, une exophtalmie inflammatoire axile non pulsatile et un segment antérieur normal. Le fond d'œil a mis en évidence une atrophie optique sans autre signe associé. Le tonus oculaire était à 30 mm hg. La TDM orbitocérebrale a confirmé l'exophtalmie et a montré l'existence d'un processus expansif intra conique bien limité prenant le produit de contraste compatible avec un hémangiome orbitaire. Le traitement a consisté en une abstinence chirurgicale et une prise en charge médicale par des antihypertenseurs locaux. Le syndrome de sturge weber est une angiomatose encéphalo-trigéminée congénitale rare qui associe un angiome cutané occupant le territoire du V1, un hémangiome lepto-méningé homolatéral et une atteinte oculaire notamment le glaucome inconstante. En fait, il s'agit d'une des principales causes de glaucome chez le sujet jeune. Le diagnostic est essentiellement clinique et doit être évoqué devant tout hémangiome facial. Les deux diagnostics différentiels sont l'angiome plan et le syndrome de Klippel-Trenaunay. Sa prise en charge doit être précoce et adaptée car il peut mettre en jeu le pronostic fonctionnel de l'œil.

**Figure 1:**
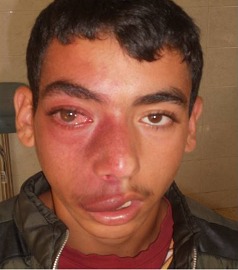
Jeune patient présentant une exophtalmie inflammatoire dans le cadre d'un syndrome de Sturge Weber

